# Editorial: Early animal development: From fertilization to gastrulation

**DOI:** 10.3389/fcell.2023.1146778

**Published:** 2023-01-26

**Authors:** Silvia L. López, M. Cecilia Cirio, Silvia Garagna

**Affiliations:** ^1^ Universidad de Buenos Aires, Facultad de Medicina, Departamento de Biología Celular e Histología/1° U.A. Departamento de Histología, Embriología, Biología Celular y Genética, Buenos Aires, Argentina; ^2^ CONICET–Universidad de Buenos Aires, Instituto de Biología Celular y Neurociencia “Prof. E. De Robertis” (IBCN), Laboratorio de Embriología Molecular “Prof. Dr. Andrés E. Carrasco”, Buenos Aires, Argentina; ^3^ Instituto de Fisiología, Biología Molecular y Neurociencias, CONICET, Facultad de Ciencias Exactas y Naturales, Universidad de Buenos Aires, Buenos Aires, Argentina; ^4^ Dipartimento di Biologia e Biotecnologie “Lazzaro Spallanzani”, Università degli Studi di Pavia, Pavia, Italy

**Keywords:** oocytes, embryogenesis, nodal signaling, T-box transcription factors, retinoic acid, MicroRNAs, β-catenin, bicaudal-C

One of the most fascinating quests in Biology is deciphering how the totipotent zygote gives rise to an animal with complex organs and systems. Several orchestrated processes must be accomplished before organogenesis. Embryos undergo a critical phase termed Maternal-to-Zygotic Transition (MZT) during which developmental control passes from mother to zygote, when a subset of maternal mRNAs and proteins is eliminated, and zygotic transcription begins. Besides, as cell number increases, cell potency decreases allowing the specification of germ layers (ectoderm, mesoderm, and endoderm), from which all tissues and organs derive. The anterior-posterior and dorsal-ventral axes are defined, setting the body plan’s coordinates. Germ layers and body axes assume their definitive spatial arrangement through gastrulation, a crucial process that drives endomesoderm internalization.

This Research Topic aimed to reflect on the recent advances in understanding the early events of embryonic development and provide new perspectives on the subject. Nine original articles and one review explore diverse aspects such as maternal cues, cell cycle regulation, morphogenetic movements, and the role of signaling pathways, transcription factors, microRNAs, and RNA binding proteins during MZT, germ layers and body axes development. The articles represent work on invertebrate and vertebrate organisms, extending to Choanoflagellates, considered the closest living relative to Metazoans.

Taking advantage of the oocyte’s animal pigmentation in the amphibian *Xenopus laevis* and the fish *Acipenser ruthenus*, Iegorova et al. present a thorough transcriptomic analysis through oocyte growth along their Animal-Vegetal axis. They identified groups of regionally distributed transcripts and proposed that degradation and *de novo* synthesis create gradient distributions for some of them. This will be a reference work for research in maternal contributions to animal development.

Nodal signaling and the T-box transcription factor Eomesodermin (Eomes) are essential for endomesoderm development in vertebrates. Two works deal with their role in zebrafish, where zygotic expression of two nodal-related genes, *ndr1* and *ndr2,* which begins dorsally and then extends to the whole blastoderm margin, is necessary for endomesoderm induction. Maternal *eomesa* transcripts are enriched vegetally, whereas the maternal transmembrane protein Huluwa (Hwa), necessary to activate the dorsalizing β-Catenin pathway, accumulates in dorsal blastomeres. Xing et al. demonstrate that zygotic *ndr1* expression depends on maternal Hwa/β-Catenin promoting *ndr1* expression on the dorsal margin; Eomesa, in the ventrolateral margin; and Nodal autoregulation being responsible for the ventral expansion of *ndr1* expression. In contrast, zygotic *ndr2* expression mainly depends on maternal Eomesa.

While *eomes* deficiency causes early lethality in mice, *eomesa* deficiency is not completely lethal in zebrafish. Talbot et al. demonstrate that the T-box transcription factor Tbx16, which is absent from Eutherians, partially compensates for *eomesa* deficiency during zebrafish endoderm formation. Moreover, *eomesa* participates in the development of the zebrafish left-right organizer (LRO), first repressing the key LRO regulator *vgll4l* and then indirectly activating it through the endoderm/LRO master transcription factor Sox32.

Two articles contributed by the same group address the role of retinoic acid (RA) during *Xenopus* embryogenesis from blastula stage and in head development. In one of them, Gur et al. show that inhibition of RA signaling at blastula stage delays gastrulation progression and migration of early involuting cells, reduces axis elongation, and alters the extracellular matrix over which cells migrate following involution, suggesting a link between RA signaling and the Wnt/PCP pathway in regulating tissue separation during gastrulation. Their observations indicate that functional RA signaling is required before gastrulation for regulating morphogenetic processes critical for normal development. In their other work, Gur et al. demonstrate that a strict balance of RA levels during embryogenesis is required for normal head development, with RA emitted by the gastrula organizer being required to prevent microcephaly. They show that the Aldehyde dehydrogenase 1 family member A3 (Aldh1a3), expressed by the anterior endomesodermal cells derived from the early gastrula organizer, is the main enzyme responsible for RA biosynthesis required for head development.

MicroRNAs regulate MZT by enhancing zygotic transcription and maternal mRNA degradation. Hu et al. show that *miR-202-3p* is critical during zebrafish MZT for embryonic viability and development. They demonstrate that in *miR-202* null embryos, abnormal development progression and apoptosis result from dysregulation of a group of three *miR-202-3p* direct target genes: *nfkiaa, perp,* and *mgll*. Their evidence points to an important role of *miR-202-3p* in the regulation of zygotic genome activation.

In the early embryos of most metazoan species, the cell cycle consists of the succession of the M and S phases without any gap. The precise timing of appearance of both G1 and G2 is scarcely known. Wong et al. developed multiple fluorescence ubiquitin cell cycle indicator reporters for the study of each cell during embryogenesis of the nematode *C. elegans*. They demonstrate that most embryonic cells skip G1 and G2 phases or have a very brief G1, with the exception of “V5QL” and “V5QR,” whose cell cycle comprises all phases.

In their review, Dowdle et al. focused on RNA binding protein Bicaudal-C (Bicc1) functions in vertebrate embryos. The *Xenopus* protein presents a vegetal-to-animal gradient where, by forming a translational repression gradient of target mRNAs, modulates the synthesis of proteins whose activities contribute to anterior-posterior polarity. A role of Bicc1 RNA regulation in controlling left-right patterning has been demonstrated in mouse, *Xenopus,* and zebrafish. The mechanisms of mRNA binding and regulation of the Bicc1 protein are described.

Echinoderms are a historically rich source for discovering mechanisms of animal embryogenesis, including the detailed study of gene regulatory networks during germ layer development. Formery et al. present a comprehensive developmental atlas for the sea urchin *Paracentrotus lividus,* covering previously neglected aspects of development as well as an updated staging scheme from fertilization to post-metamorphic juvenile stages, providing an important reference tool for the scientific community.

The emergence of the primitive gut is one of the decisive events leading to the origin of Metazoans, but the existence of a common evolutionary developmental mechanism for this organ remains uncertain. Nguyen et al. employed hydrodynamic mechanical strains, reminiscent of the soft marine flow context where pre-metazoan colonies evolved, to study tissue invagination in the diploblastic metazoan *Nematostella vectensis* (cnidaria) and the multicellular choanoflagellate *Choanoeca flexa*. They show that hydrodynamic stimulation activates tissue invagination *via* Myosin-II-dependent mechanotransduction. Like in bilaterian animals, invagination in *N. vectensis* depends on endomesoderm specification *via* biomechanical-based β-Catenin phosphorylation. They propose that in early Metazoans, primitive gut formation, tissue invagination/gastrulation, and endomesoderm specification may have been initiated by a mechanotransduction mechanism.

We hope that the aspects covered here will reflect on the recent advances in understanding the early events of embryonic development and provide new resources for contemplating this long-standing question of how multicellular organisms evolved and are built.

